# Microstructure and Phase Composition of Cold Isostatically Pressed and Pressureless Sintered Silicon Nitride

**DOI:** 10.1186/s11671-016-1365-1

**Published:** 2016-03-15

**Authors:** O. A. Lukianova, V. V. Krasilnikov, A. A. Parkhomenko, V. V. Sirota

**Affiliations:** Belgorod National Research University, 85, Pobedy Str., 308015 Belgorod, Russia; Institute of Hydrobiology of the National Academy of Sciences of Ukraine, 2, Geroyev Stalingrada ave., 04210 Kyiv, Ukraine

**Keywords:** Silicon nitride, Microstructure, Grain size, Phase composition

## Abstract

The microstructure and physical properties of new Y_2_O_3_ and Al_2_O_3_ oxide-doped silicon nitride ceramics fabricated by cold isostatic pressing and free sintering were investigated. The phase composition of produced material was also studied by X-ray diffraction at room and elevated temperature. The fabricated ceramics featured a microstructure of Si_5_AlON_7_ grains with a fine-grained α-Si_3_N_4_ with a small amount of Y_2_SiAlON_5_. Described ceramics is attractive for many high-temperature structural applications due to beneficial combination of fine-grained structure with improved mechanical properties and small weight loss.

## Background

Silicon nitride is one of the most promising structural materials for high-temperature applications because of its excellent strength and toughness at elevated temperatures, good thermal shock resistance, low coefficient of thermal expansion, and chemical stability [1–3]. Silicon nitride ceramics are frequently used as structural materials especially for high-temperature engineering applications. The high-temperature properties of Si_3_N_4_-based ceramics strongly depend on the oxide additives used for the densification. However, its densification is rather difficult by classical sintering process due to the strongly covalent in Si–N bonds which results in low self diffusivity [4]. Such oxide additives as MgO, Al_2_O_3_, Y_2_O_3_, and Al_2_O_3_ + Y_2_O_3_ combination are considered as the most commonly used and ideal additives for Si_3_N_4_ ceramics due to their high melting point and because of the possibility to control the α→β phase transformation rates of the Si_3_N_4_, the aspect ratio of the β-Si_3_N_4_, and the grain growth anisotropy. These additives also lead to high mechanical properties at room temperature as well as at elevated temperatures [3]. The use of rare-earth oxide additives to provide a liquid phase for sintering is therefore required for obtaining high-density Si_3_N_4_ [5, 6]. The control of the grain size and its distribution is one of the most important issues in the processing of Si_3_N_4_ ceramics. Particularly, the design of duplex microstructure containing a few large elongated β-Si_3_N_4_ grains is quite important to ensure high fracture toughness [7–9]. One of the major problems of the silicon nitride processing is the high cost of such commercial methods as hot isostatic pressing (HIP), spark plasma sintering (SPS), and gas pressure sintering (GPS). Thus, it is important to find new and cheaper alternative processing methods while a relatively high strength can be acquired. To overcome this problem, we have developed a novel process involving cold isostatic pressing (CIP) and pressureless sintering in a nitrogen atmosphere.

The purpose of this work is to study the features of the microstructure and phase composition of new CIPed and pressureless sintered silicon nitride ceramics with Al_2_O_3_ and Y_2_O_3_ oxide.

## Methods

**Fig. 1:**
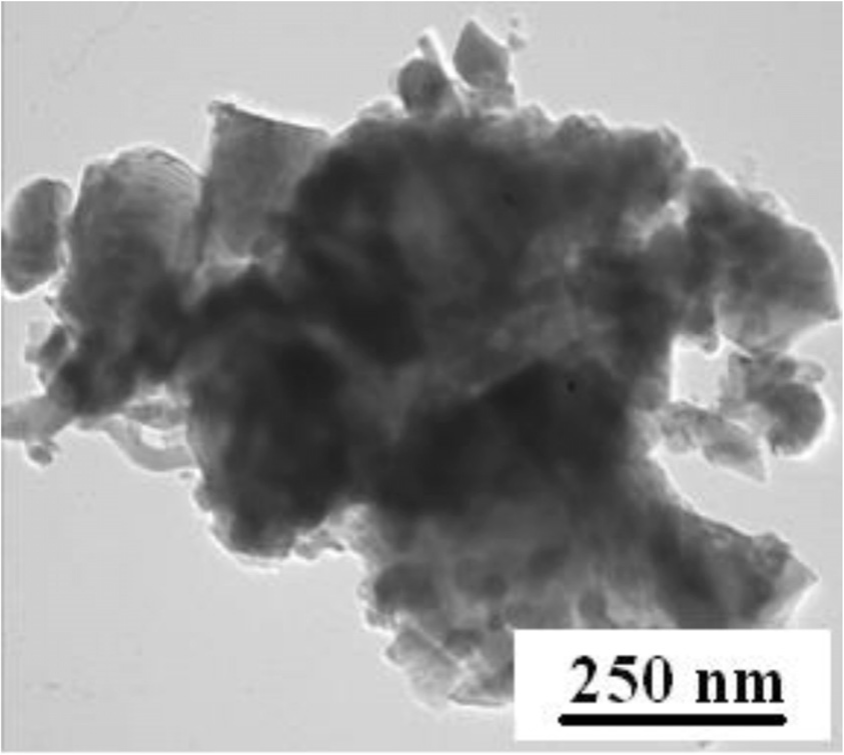
TEM image of the initial commercial silicon nitride powder

Commercial α-Si_3_N_4_ powder (Starck, grade M11) was used as the starting powder, and Y_2_O_3_ (grade B) and Al_2_O_3_ (A 16 SG Grade, Alcoa) were used as the sintering additives. The powder mixture was composed of 95 wt% α-Si_3_N_4_ powder and 15 wt% sintering additives (Table 1). The powder mixture was milled in an attritor mill for 20 min. After milling, the powder mixture was cold isostatically pressed to rectangular bars at 200 MPa (EPSI CIP 400 B-9140 press). The green bodies were sintered in a vacuum furnace (Nabertherm VHT 8/22-GR) under 0.1 MPa in a nitrogen atmosphere at 1650 °C (Table 1). For more details and properties, see [10–12]. Figure 1 shows the agglomerate of the initial commercial silicon nitride powder observed by TEM.

**Table 1:** Chemical composition of the starting materials and physical properties

Si_3_N_4_, wt%	Al_2_O_3_ wt%	Y_2_O_3_ wt%	Pressure (CIP), MPa	Powder bed	Temperature, °C
85	9	6	200	Si_3_N_4_	1650

Phase composition of the sintered samples was determined by the X-ray diffraction method (XRD, Rigaku Ultima IV diffractometer; Cu Kα—emission (radiation), Ni—filter). A scan rate of 10 °C/min was used to record the diffraction patterns in 2θ range between 10 and 60 °C. XRD analyses were carried out using a Rigaku Ultima IV automated diffractometer. The sintered material was analyzed in the solid form.

The microstructure was characterized by scanning electron microscopy (SEM) and transmission electron microscopy (TEM). Structural characterization was performed using an FEI Quanta 200-3D (FEI Company, Hillsboro, OR) and Quanta 600 FEG (FEI Company, Hillsboro, OR) scanning electron microscopes. Since the silicon nitride material under investigation was non-conducting, it was necessary to coat it with a thin layer of carbon to prevent surface charging during examination. One specimen from batch was examined in a transmission electron microscope (JEOL-2100) at 200 kV. Foils for TEM were prepared from slices hand ground and finally ion-polished until perforation.

Thermogravimetric analysis (TGA)/differential thermal analysis (DTA) experiments were performed using an equipment STA 503 BÄHR model at a heating rate of 10 °C/min.

For surface examination, specimens were mechanically polished.

## Results and Discussion

**Fig. 2:**
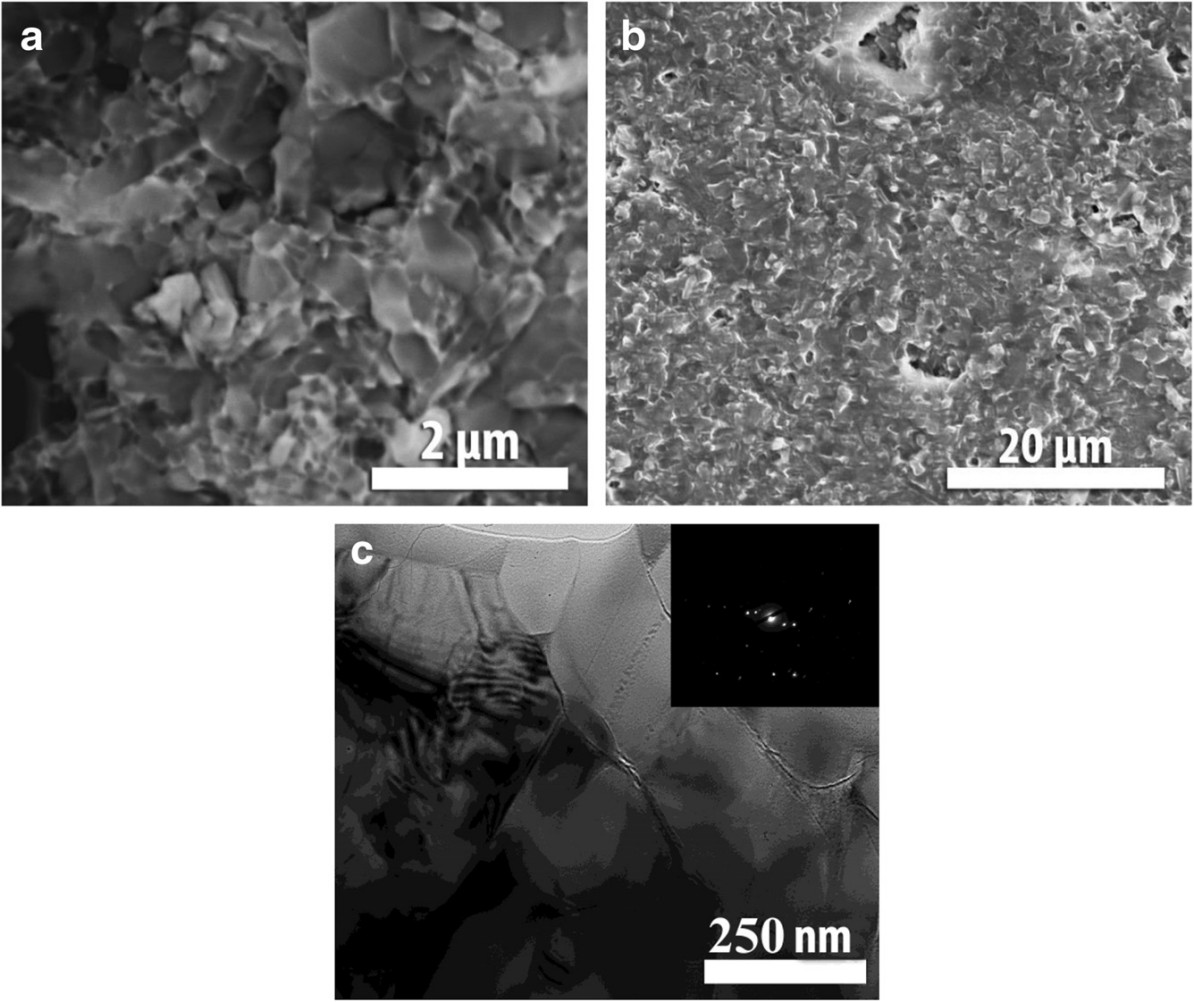
Microstructures of Si_3_N_4_ sintered with yttrium and aluminum oxide additives. **a** Surface by SEM. **b** Fracture by SEM. **c** Thin specimen by TEM

A lot of researches and developments indicated that high fracture toughness has been attributed to the elongated β-Si_3_N_4_ grains and weak grain boundary, which favors crack bridging and deflection. It was reported that the materials having microstructure with the finest and coarsest elongated β-grains showed the high value of fracture toughness, whereas materials with the microstructure with the mixture of elongated thin and thick β-grains had lower facture toughness [13]. Figure 2 shows a microstructure of a polished specimen. Produced ceramics is characterized by the bimodal character of microstructure with a grain size distribution from 300 to 800 nm. The microstructure generally consisted of large and small equiaxed grains.

The density of our material was 2.94 g/cm^3^, the flexural strength was 280 MPa, the Young’s modulus was 214 GPa, the microhardness was 1375 HV, and open porosity was 0.1 % as it described in our previous work [10]. Abe et.al described the correlation of CIP condition with structure and properties of both green and sintered bodies [5]. Hotta et al. described the microstructure and fracture strength of CIPed silicon nitride [6]. Sintered in a nitrogen atmosphere for 3 h, materials exhibit high mechanical properties, in particular, a high bending strength 607 and 686 MPa, respectively, and fracture toughness 5.6 MPa/m^−1/2^ [5, 6]. Furthermore, it should be noted that reported high strength ceramics have the same type of microstructure comparing with our ceramics.

**Fig. 3:**
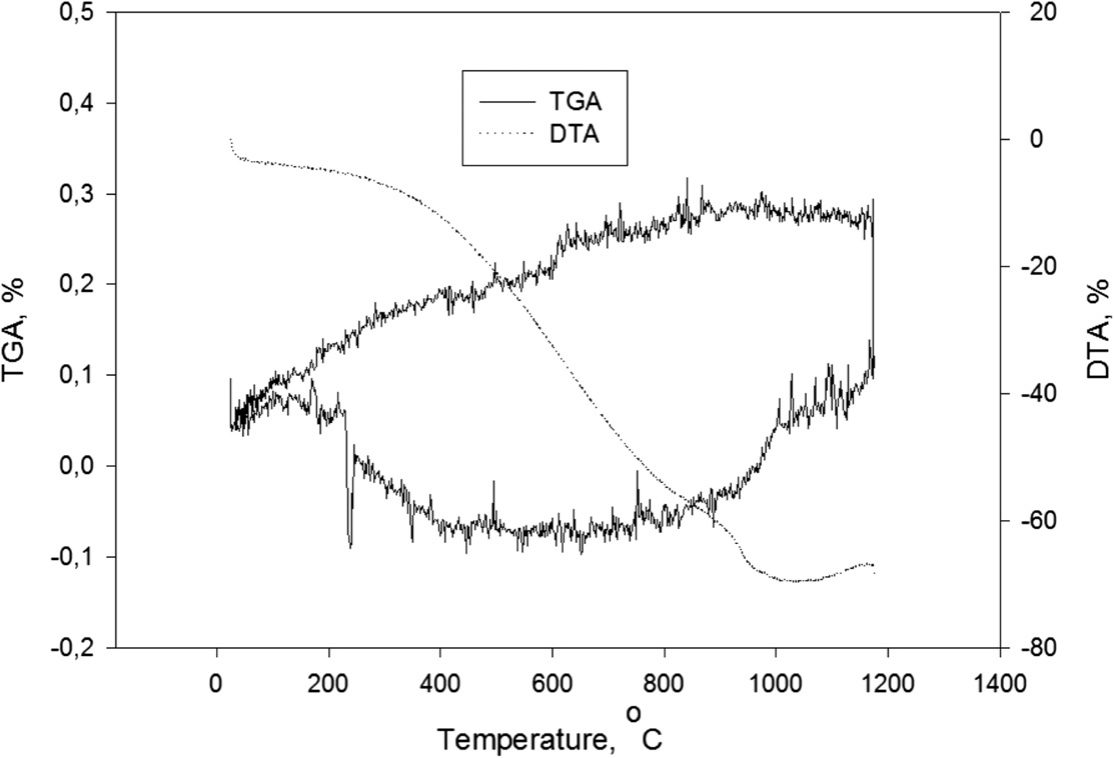
Thermogravimetry-differential thermal analysis curves of Si_3_N_4_

Figure 3 presents the results of thermal analysis. The sample firstly starts losing weight at about 200 °C and ends at about 400 °C. The weight loss of the present ceramics was 0.003 % (Table 2).

**Table 2:** Physical properties

Mass loss, %	Flexural strength, MPa [10]	*ρ*, g/cm^3^ [10]	Open porosity, % [10]
0.003	280	2.94	0.01

Matovic and co-authors investigated pressureless sintered Si_3_N_4_ with the Li_2_O–Y_2_O_3_ additives. The density of this ceramics ranged from 83.8 to 98.0 % of the theoretical density depending on the amount and type of oxide additives. The weight loss was 3.7 and 4.1 %, respectively [8]. Cinibulk and Thomas described the same type of structure for sintered at 1850 and 1900 °C silicon nitride with a YSiAlON glass. β-Y_2_Si_2_O as a secondary phase was observed after the heat treatment at 1350 °C, while at a higher temperature of 1450 °C, primarily YSiO_2_N and Y_4_Si_2_O_7_N_2_ in addition to small amounts of Y_2_SiOs were found [9].

**Fig. 4:**
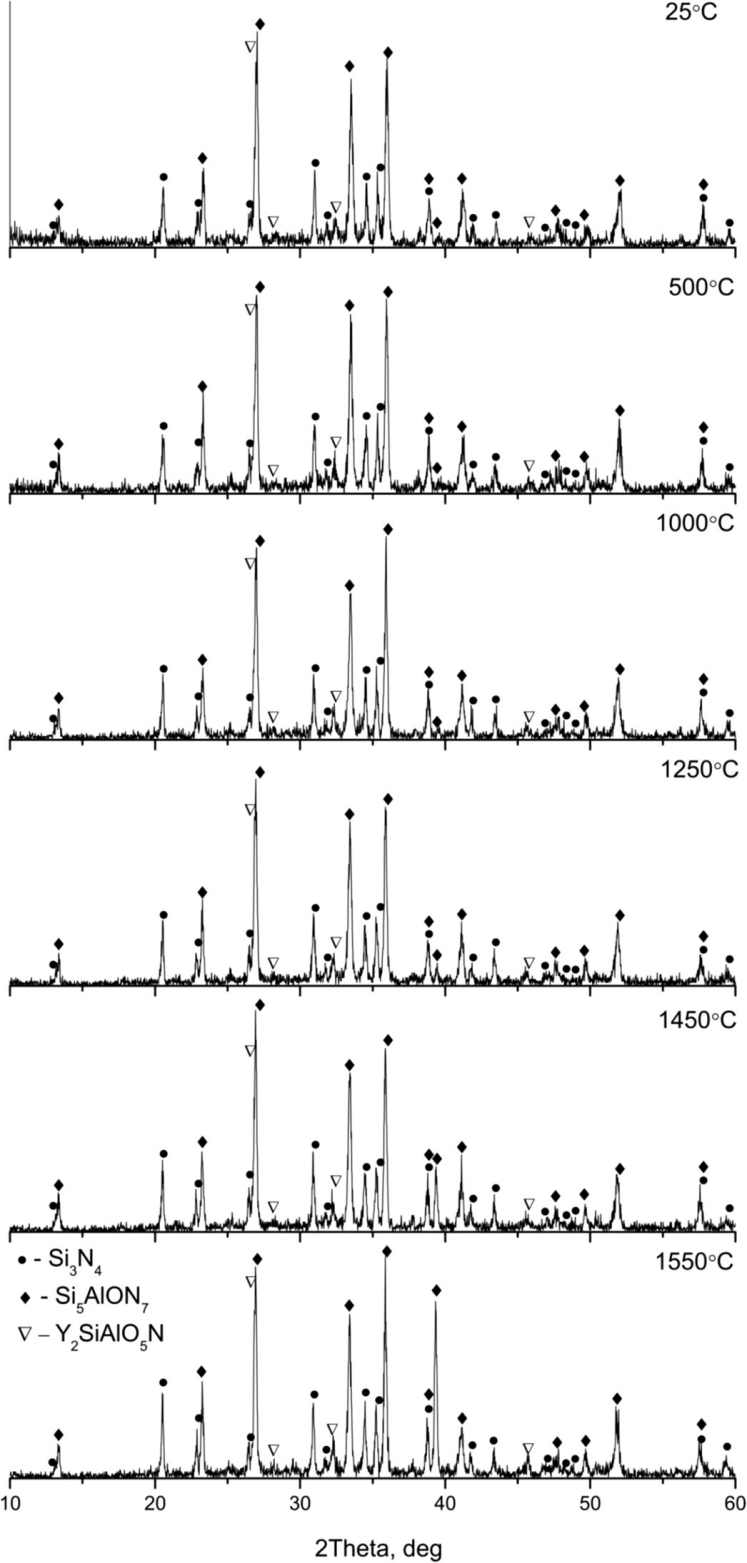
High-temperature XRD

High-temperature XRD patterns of the sintered samples are shown in Fig. 4. The XRD analyses revealed β-Si_5_AlON_7_ as a major phase, α-Si_3_N_4_ phase, and Y_2_SiAlON_5_ as a secondary phase in the produced ceramics. It should be noted that all specimens could be sintered to higher than 95 % of theoretical density. Free Si was not observed. Ling and coauthors described pressureless sintered silicon nitride with MgO as an additive. It was only β-silicon nitride that existed in the as-sintered samples. Neither MgO or Y_2_O_3_ nor other crystalline phase was detected [6].

Yang et al. described porous silicon nitride with 5 wt% different rare-earth oxides sintering additives (Y, La, Lu, and Eu) fabricated by pressureless sintering. A β-Si_3_N_4_ phase was only found by XRD analysis. This ceramics showed a typical bimodal microstructure with a large elongated β-Si_3_N_4_ grains and small β-Si_3_N_4_ matrix grains. The porosity of the described material varied from 48 to 54 %, the bending strength ranged from 150 to 188 MPa, the fracture toughness varied from 1.6 to 3.1 MPa/m^−1/2^, and the weight loss varied from 4 to 7 % [7]. Chen et al. also described silicon nitride ceramics with controlled porosity from 42 to 63 % and relatively high flexural strength from 50 to 120 MPa [14]. Luo and co-authors described 90 and 95 % dense Si_2_N_2_O/β-Si_3_N_4_ composites fabricated by cold isostatic pressing and sintering at 1700 and 1800 °C. This material consists of Si_2_N_2_O and β-Si_3_N_4_ phases and has a grain size of 2 and 0.5 μm, respectively, depending on the sintering temperature [15]. Sillapasa and others investigated uniaxially pressed and further cold isostatically pressed pre-sintered at argon atmosphere and then finally sintered in a nitrogen atmosphere silicon nitride. The bulk density of described material ranged from 2.18 to 2.61 g/cm^3^, the porosity varied from 5.78 to 21.30 %, the Young’s modulus changed from 121.5 to 198.6 GPa, the bending strength ranged from 104.0 to 250.7 MPa, and the microhardness varied from 403 to 971 HV [16].

The content of all phases changes insignificantly with an increase in the temperature from 20 to 1550 °C (Table 3). In particular, the content of Si_5_AlON_7_ varied from 64.1 to 72.7 %, the content of α-Si_3_N_4_ ranged from 25.2 to 33.4 %, and the amount of the Y_2_SiAlON_5_ varied from 2.1 to 2.5 %. Gonon with co-authors described in detail characteristics of the Y_2_SiAlON_5_ [17]. Further increase in the testing temperature up to 1500 °C is accompanied with a decrease in the β-SiAlON content up to 68.4 %. Some physical properties of the free sintered magnesium oxide doped ceramics were described in Sirota et al. [18].

**Table 3:** Phase composition

Temperature, °C	Phase, %		
	Y_2_SiAlON_5_	Si_5_AlON_7_	α-Si_3_N_4_
25	2.3	71.5	26.2
500	2.1	72.3	25.6
1000	2.1	72.7	25.2
1250	2.1	71.5	26.4
1450	2.5	64.1	33.4
1550	2.3	68.4	29.3

Tables 2 to 4 summarize the variation of density, mass loss, phase content, and lattice parameter of the specimen after sintering. The data for the specimens referred from the previous paper are also listed in Table 2.

**Table 4:** Phase characteristics

	Space group	Lattice constant, *Å*	
		*a*	*c*
Y_2_SiAlON_5_	P6_3_/m (176)	3.818	9.980
Si_5_AlON_7_	P6_3_ (173)	7.629	2.927
α-Si_3_N_4_	P31c (159)	7.753	5.624

## Conclusions

The fabrication of a fine-grained silicon nitride ceramics by cold isostatic pressing and pressureless sintering was reviewed. Commercial submicronic powders were used as raw materials. Al_2_O_3_ and Y_2_O_3_ additives lead to moderately high density and low weight loss.

The results can be summarized as follows:

1. The obtained ceramic has a fine-grained microstructure with equiaxed grains with the average grain size that ranged from 0.3 to 0.8 μm.
2. Investigated ceramics mainly consists of α-Si_3_N_4_ and Si_5_AlON_7_ phases and a small amount of the Y_2_SiAlON_5_ phase. Phase composition does not change in a wide temperature range up to 1500 °C.
3. Relatively high mechanical properties are successfully combined with the absence of porosity and low weight loss. Therefore, the proposed ceramics can be effectively used in many areas of high temperature applications.

## Abbreviations

CIP: cold isostatic pressing
DTA: differential thermal analysis
HIP: hot isostatic pressing
SEM: scanning electron microscopy
SPS: spark plasma sintering
TEM: transmission electron microscopy
TGA: thermal gravimetric analysis
XRD: X-ray diffraction
